# Case comparison of nursing homes during COVID-19: Lessons for future infectious disease outbreaks

**DOI:** 10.1093/geroni/igaf122.4393

**Published:** 2025-12-31

**Authors:** Amy Vogelsmeier, Lori Popejoy, Steven Miller, Melissa Taylor, Linda Anderson

**Affiliations:** University of Missouri - Columbia, Columbia, Missouri, United States; University of Missouri - Columbia, Columbia, Missouri, United States; University of Missouri Sinclair School of Nursing, Columbia, Missouri, United States; University of Missouri - Columbia, Columbia, Missouri, United States; University of Missouri - Columbia, Columbia, Missouri, United States

## Abstract

U.S. nursing homes (NH) were affected by the COVID-19 pandemic with over 2.2 million confirmed cases and over 150,000 resident deaths. This analysis explored differences in NHs’ response during COVID-19 including activities that may have influenced resident outcomes and was part of a larger mixed-methods study to understand NHs response to COVID-19. Interviews with leaders, infection preventionists, staff, residents/families from 24 Midwestern NHs were analyzed using directed content analysis and summarized into individual NH cases. We selected 10 NHs for case-comparison based on their 2020 Nursing Home Safety Net all-cause mortality Data: five high mortality NHs (HMNH) and five low mortality NHs (LMNH). Most HMNHs were non-corporate/standalone (n = 4, 80%) and urban (n = 3, 60%). Most LMNHs were corporate owned (n = 3, 60%) and rural (n = 3, 60%). Bed size (61-120) was similar (HMNH n = 3, 60%; LMNH n = 4, 80%). No differences were noted for leaders or medical director involvement. LMNHs had more RN infection preventionists compared to HMNH (n = 4, 80%, n = 2; 40%, respectively). Implementation of regulatory-based guidelines e.g. cohort, isolation, quarantine, PPE use, testing/reporting, visitor restrictions were similar, however, HMNHs experienced greater challenges with PPE access and longer test result turnaround times. Both groups used agencies for staffing shortages, however, LMNHs had more consistent processes for managing agency staff. Both groups promoted vaccinations except one HMNH encouraged medical/religious exemptions with the goal of turnover reduction. Both groups shared concerns about the impact of COVID-19 on staff/residents’ health/well-being. Differences in how NHs responded to COVID-19 and implications for future infectious disease outbreaks will be described.

